# Preliminary Report of Method for Estimating in Vivo the Germicidal Activity of Antiseptics

**Published:** 1919

**Authors:** 


***Preliminary Report of Method for Estimating in Vivo the Germicidal Activity of Antiseptics.*** By JOSEPH A. PERKINS. *Annals of Surgery*, September 1918.

The method of estimating and graphically recording the bacterial contents of a wound with the microbe charts works out well clinically, but the results are, at best, very crude.

In order to reduce as far as possible the element of personal equation, the work has been done by one man. The inoculations were made from the same part of the surface of the wound; the attempt was made to get a uniform-sized drop. The drop obtained was inoculated at the bedside in 2 cc. of plain bouillon, and the bouillon suspension, undiluted, was immediately poured over an agar-agar plate. The plate was then taken to the laboratory, placed in an incubator, and kept at 37 degrees C. At the end of 24 hours the colonies were counted macroscopically and recorded,

In the first series of charts, using the dichloramine-T in oil of eucalyptol in 24 hours, following the initial drop there is a slow, gradual rise in the curve, which shows, we believe, a continual activity of the germicidal properties of the antiseptic lasting from 14 to 16, and 18 and 20 hours, respectively, in the three cases.

Then with hypochlorite solution there is again the initial drop, followed this time by an immediate rise to infinity, in one case within an hour, in the other, two hours, showing the admittedly short time the antiseptic is active.

Using the hypochlorite solution every two hours, there is each time the initial drop, but each time before the following two hours are up there is the rebound back to infinity; and after six instillations of the hypochlorite solution, twelve hours' treatment, the bacterial count is still above infinity, as against the comparatively low count 100, and 62, in the same cases twelve hours after a single treatment with dichloramine-T.

In the last two charts, using dichloramine-T, this time in the chlorcosane solvent, we have the initial drop followed by the gradual rise in the count, showing a germicidal activity lasting throughout the full 22 hours.

We feel that through this method some idea can be obtained of the comparative strength of antiseptics, and the length of time during which they are active when applied to human tissues in the presence of infection.

